# Two-in-one strategy: a remineralizing and anti-adhesive coating against demineralized enamel

**DOI:** 10.1038/s41368-020-00097-y

**Published:** 2020-09-29

**Authors:** Ailin Hou, Jun Luo, Min Zhang, Jianshu Li, Wenlin Chu, Kunneng Liang, Jiaojiao Yang, Jiyao Li

**Affiliations:** 1 State Key Laboratory of Oral Diseases & National Clinical Research Center for Oral Diseases & Department of Cariology and Endodontics, West China Hospital of Stomatology, Sichuan University, Chengdu, China; 2 College of Polymer Science and Engineering, Sichuan University, Chengdu, China; 3 College of Polymer Science and Engineering & State Key Laboratory of Polymer Materials Engineering, Sichuan University, Chengdu, China; 4 State Key Laboratory of Oral Diseases & National Clinical Research Center for Oral Diseases, West China Hospital of Stomatology, Sichuan University, Chengdu, China

**Keywords:** Biomineralization, Biomimetic synthesis, Biomineralization, Biomimetic synthesis

## Abstract

Tooth enamel is prone to be attacked by injurious factors, leading to a de/remineralization imbalance. To repair demineralized enamel and prevent pulp inflammation caused by biofilm accumulation, measures are needed to promote remineralization and inhibit bacterial adhesion on the tooth surface. An innovative material, poly (aspartic acid)-polyethylene glycol (PASP-PEG), was designed and synthesized to construct a mineralizing and anti-adhesive surface that could be applied to repair demineralized enamel. A cytotoxicity assay revealed the low cytotoxicity of synthesized PASP-PEG. Adsorption results demonstrated that PASP-PEG possesses a high binding affinity to the hydroxyapatite (HA)/tooth surface. In vitro experiments and scanning electron microscopy (SEM) demonstrated a strong capacity of PASP-PEG to induce in situ remineralization and direct the oriented growth of apatite nanocrystals. Energy dispersive X-ray spectroscopy (EDS), X-ray diffraction analysis (XRD) and Vickers hardness tests demonstrated that minerals induced by PASP-PEG were consistent with healthy enamel in Ca/P ratio, crystal form and surface micro-hardness. Contact angle tests and bacterial adhesion experiments demonstrated that PASP-PEG yielded a strong anti-adhesive effect. In summary, PASP-PEG could achieve dual effects for enamel repair and anti-adhesion of bacteria, thereby widening its application in enamel repair.

## Introduction

Tooth enamel is the hardest mineralized tissue in the human body, consisting of the outermost layer of the dental crown.^1^ Generally, the demineralization and remineralization of enamel maintain a dynamic equilibrium. Attacked by acids or caries, the enamel lesions come to form as a result of the de/remineralization imbalance.^2,3^ Once the enamel is damaged, whether by cariogenic bacteria, chemical acids, or mechanical stress, clinical treatments are needed to repair the demineralized enamel since it is a non-living tissue and is not able regenerate.^1,4^ Conventional interventions in clinical practice mainly consist of the usage of topical fluoride and restorative treatment. Nevertheless, repeated application of fluoride is needed to maintain sustained topical concentrations,^5^ which may increase the incidence of dental and skeletal fluorosis.^6^ For restorative treatment, the main flaws are the ageing of resin composite resin and the propensity of new caries to form at the margins of restorations if the causes of the disease are not removed.^7^ In addition, this treatment requires the sacrifice of surrounding healthy tooth tissue.^8^

Biomimetic mineralization is stipulated as the organic molecules to induce the formation of inorganic crystals with specific structures and properties. Bioinspired mineralization has provided a new versatile technology in the fields of dental restoration.^9^ In nature, many extracted proteins (e.g., tuffien and enamelin) associated with mineralized tissue have a high content of acidic moieties that promote hard tissue generation, such as aspartic acid (Asp).^10–12^ These acidic residues are considered to be an important contributor to HA binding and biomineralization induction.^10,12^ To mimic biomineralization, a number of studies have been performed using poly (aspartic acid) (PASP),^13^ (poly)peptides^13–15^ or other polymers containing Asp^16^ as analogs of such mineralization proteins to repair demineralized enamel.

On the other hand, due to the poor antifouling properties of currently available biomimetic mineralization materials, oral bacteria adhere to the enamel surface easily to form dental plaque biofilms, which produce acids and other toxins, leading to pulp infection. Therefore, developing a convenient technique combining remineralization and antifouling is necessary but challenging to repair demineralized enamel. Polyethylene glycol (PEG) is a nontoxic and highly hydrophilic biocompatible polymer. It is considered the “gold standard” of antibiofouling polymers and has been widely used in biomedical systems.^17,18^ When tethered to surfaces, PEG acts as a brush-like barrier^19^ to inhibit nonspecific adsorption of microorganisms, such as PEGylated phase-transitioned lysozyme nanofilm for resisting the attachment of oral bacteria,^20^ and (PEG)-based copolymer reducing the accumulation of cariogenic bacteria on dental surfaces.^21^

In the present study, we aimed to construct a bifunctional material by combining the anti-adhesive property of PEG and the remineralization property of PASP and applied it to the repair of demineralized enamel for the first time. We hypothesize that PEG functionalized with PASP offers a new approach to repairing demineralized enamel by suppressing bacterial adhesion and inducing in situ mineralization on acid-etched tooth surfaces.

## Results

### Structural characterization and cytotoxicity evaluation of PASP-PEG

**Fig. 1:**
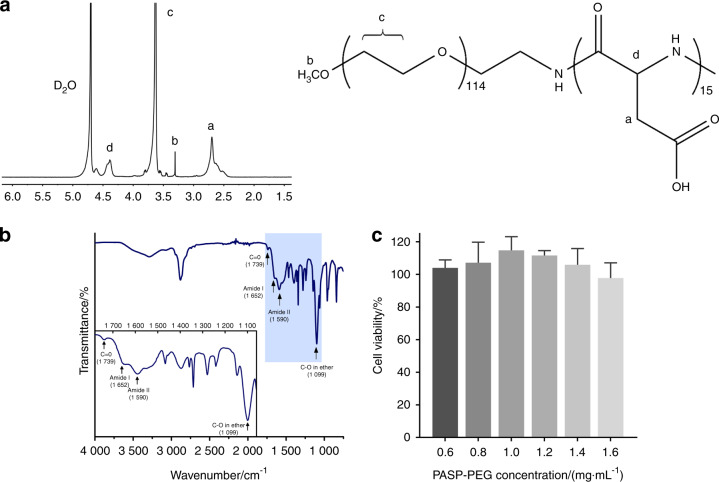
Characterization of PASP-PEG. **a** ^1^H NMR spectrum of PASP-PEG. **b** ATR-FTIR spectrum of PASP-PEG. The inset is the enlargement of characteristic peaks assigned to C═O, amide I, amide II and C−O stretching. **c** The cell viability of human oral keratinocytes (HOKs) treated with PASP-PEG for 24 h. HOKs displayed superior viability in the range of 97.75% to 114.79% (mean ± SD, *n* = 5)

The chemical structures of PASP-PEG were confirmed by ^1^H NMR (Fig. 1a) and ATR-FTIR (Fig. 1b). As shown in Fig. 1a, the ^1^H NMR spectrum of PASP-PEG shows several distinct signals, which can be assigned as follows: δ = 2.70 ppm, the signal of methylene protons (–CH_2_–) in the side-chains of Asp (a); δ = 3.31 ppm, the signal of methyl protons (–CH_3_) in the end-cap of PEG (b); δ = 3.63 ppm, the signal of the methylene protons (–CH_2_–) of the PEG chain (c); δ = 4.39 ppm, the signal of methine protons (–CH–) in PASP backbone (d). The methyl groups and methine protons demonstrated that the degree of polymerization of Asp in the block copolymer was 15. In the ATR-FTIR spectrum of PASP-PEG (Fig. 1b), the vibration peak at 1 739 cm^−1^ was assigned to the carbonyl group (C═O). Two characteristic peaks at 1 652 cm^−1^ (amide I) and 1 590 cm^−1^ (amide II) demonstrated the ring-opening of succinimide groups and the formation of amido bonds. The vibration peak at 1 099 cm^−1^ was attributed to C−O stretching, characteristic of the ether bonds of PEG. Figure 1c shows the cytotoxicity of PASP-PEG at various concentrations from 0.6 to 1.6 mg·mL^−1^. The cells treated with PASP-PEG displayed superior viability in the range of 97.75% to 114.79%, which was of no significant difference compared with the control group.

### Adsorption capability of PASP-PEG on HA powders and tooth enamel

**Fig. 2:**
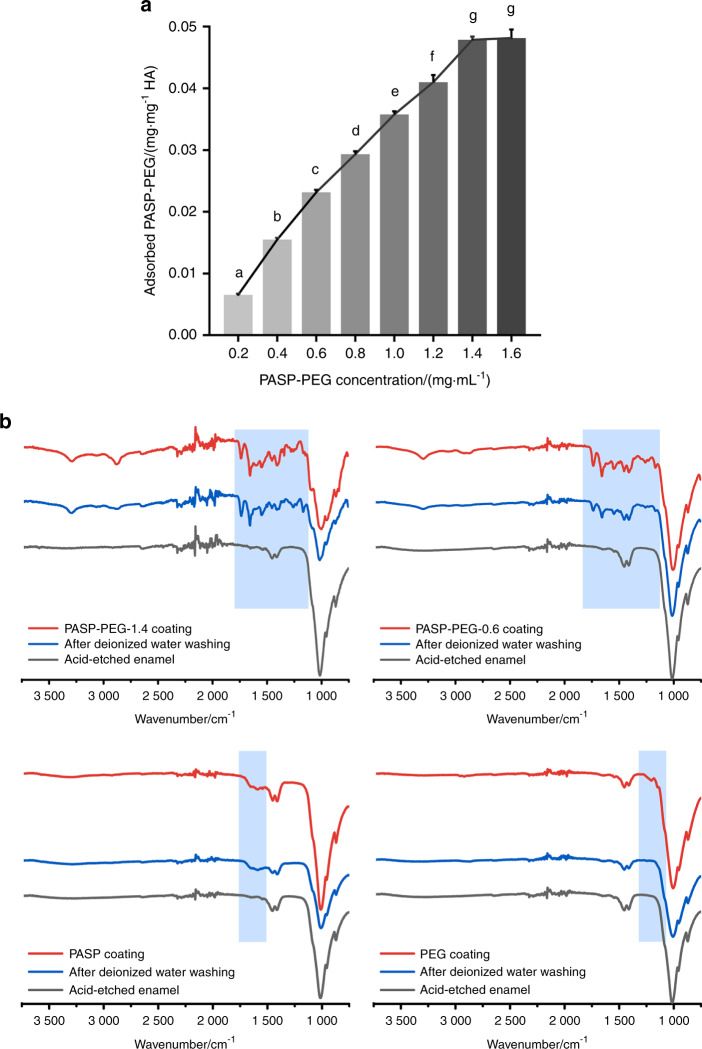
Adsorption of PASP-PEG on HA powder and tooth enamel. **a** Adsorption isotherm of PASP-PEG (0.2 mg·mL^−1^ to 1.6 mg·mL^−1^) on 25 mg HA powder (mean ± SD, *n* = 5). Dissimilar letters indicate significantly different values (*P* < 0.01). **b** ATR-FTIR spectra of acid-etched tooth enamel, PASP-PEG-1.4, PASP-PEG-0.6, PASP and PEG coating enamel and after washing with deionized water. The characteristic peaks of PASP-PEG-1.4, PASP-PEG-0.6, and PASP remained strong after washing with deionized water

The adsorption curve of PASP-PEG showed that the optimum absorption wavelength was 288 nm (Supplementary Fig. S1a), which was employed to define the standard curve (Supplementary Fig. S1b). Figure 2a shows the adsorption isotherm of PASP-PEG with different concentrations of HA powder (25 mg, mean ± SD, *n* = 3). It can be observed that the adsorbed PASP-PEG on HA increased rapidly at first (concentration of PASP-PEG from 0.2 mg·mL^−1^ to 1.4 mg·mL^−1^) and then approached saturation (concentration of PASP-PEG from 1.4 mg·mL^−1^ to 1.6 mg·mL^−1^). The saturated adsorption amount of PASP-PEG was (0.0512 ± 0.0065) mg·mg^−1^ HA when the concentration was 1.6 mg·mL^−1^, which was not significantly different from (0.0479 ± 0.0004) mg·mg^−1^ HA when the concentration was 1.4 mg·mL^−1^. Thus, 1.4 mg·mL^−1^ PASP-PEG was chosen for further experiments. Figure 2b displays the adsorption results of PASP-PEG-1.4, PASP-PEG-0.6, PASP and PEG on acid-etched enamel. After being coated by PASP-PEG, the characteristic peaks at 1 738 cm^−1^ (C═O), 1 649 cm^−1^ (amide I), 1 590 cm^−1^ (amide II) and 1 098 cm^−1^ (C−O) appeared on samples of both the PASP-PEG (1.4 mg·mL^−1^) (PASP-PEG-1.4) and PASP-PEG (0.6 mg·mL^−1^) (PASP-PEG-0.6) groups. The peaks at 1 655 cm^−1^ and 1 591 cm^−1^ were assigned to amido bonds for samples of the PASP (0.65 mg·mL^−1^) (PASP) group, and the signal at 1 133 cm^−1^ was assigned to C−O stretching for samples of the PEG (0.75 mg·mL^−1^) (PEG) group. After deionized water washing, the peaks (1 738, 1 649, 1 590 and 1 098 cm^−1^) of PASP-PEG and peaks (1 655 and 1 591 cm^−1^) of PASP on the samples remained strong. However, the characteristic peak at 1 133 cm^−1^ (C−O) of samples treated with PEG disappeared after washing with deionized water.

### Remineralization of human tooth enamel induced by PASP-PEG in vitro

**Fig. 3:**
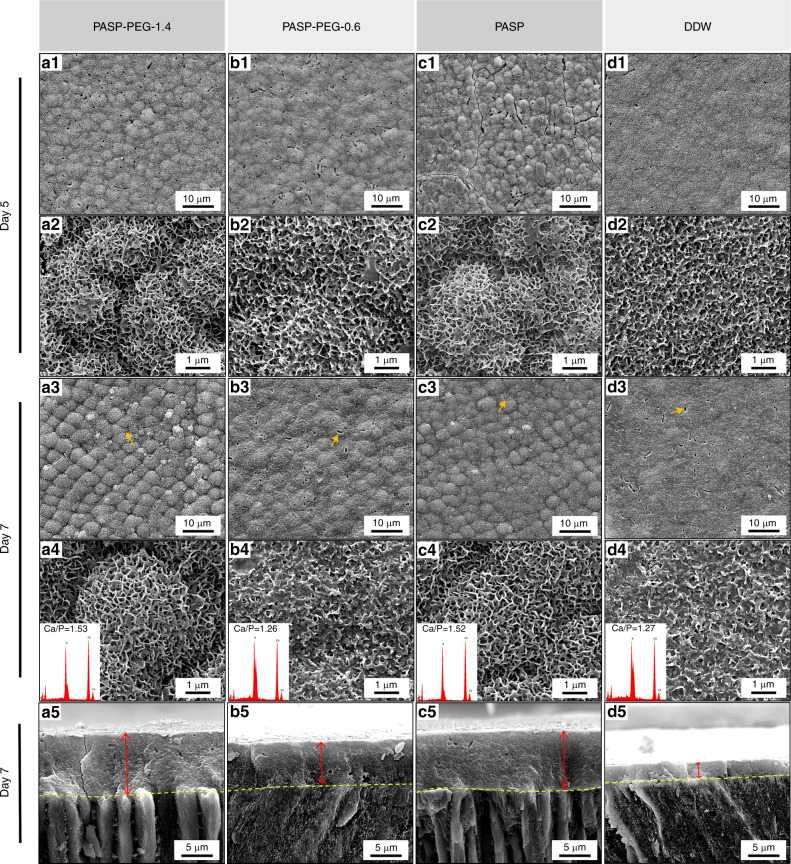
PASP-PEG-induced remineralization of enamel. SEM images of acid-etched enamel treated with PASP-PEG-1.4, PASP-PEG-0.6, PASP and DDW after immersion in 1.5× SBF for 5 d (a1–d1, a2–d2; a2–d2 are enlargements of a1-d1) and 7 d (a3–d3, a4–d4; a4–d4 are enlargements of a3–d3). The arrows in a3–d3 show gel-like ACP. The insets in a4-d4 are the EDS spectra of the whole area of a4–d4. a5–d5 SEM images of the cross-section of acid-etched tooth enamel treated with PASP-PEG-1.4, PASP-PEG-0.6, PASP and DDW after being soaked in 1.5× SBF for 7d

The acid-etched tooth enamel samples coated with PASP-PEG-1.4, PASP-PEG-0.6, PASP and deionized water (DDW) were incubated in 1.5 × SBF for different time periods. SEM images of untreated acid-etched tooth enamel are shown in Supplementary Fig. S2. The interprismatic area of tooth enamel was eroded, and the enamel prism structure could be clearly observed. After incubation in 1.5 × SBF, the crystals on the enamel surface were regenerated with increasing incubation time (Fig. 3). Fig. 3a1–d1 shows that samples of all groups were completely covered by a layer of newly formed minerals, and the exposed prism and interprismatic area were invisible after incubation for 5 d. Additionally, the regenerated crystals on the surface showed differences in the morphological features of different groups. The surfaces of samples treated with PASP or PASP-PEG presented distinctive prism-like structures at low magnification (Fig. 3a1–c1), with the appearance of prism-like structures being enhanced in the PASP-PEG-1.4 and PASP groups. However, the enamel surfaces of the DDW group showed no prism-like structures, resulting in a relatively flat and porous renewed layer (Fig. 3d1). Fig. 3a2–d2 exhibit the corresponding high magnification SEM images of a1-d1. Wave-like microplates highly interconnected with each other to form porous flower cluster crystals on samples from the PASP-PEG-1.4 and PASP groups, which looked similar to a cross section of honeycomb or a sponge (Fig. 3a2 and c2). Compared with these two groups, the new minerals aggregated to form microplates with much smaller size and relatively nonuniform shape, more like sparse whiskers scattering on samples from the PASP-PEG-0.6 and DDW groups (Fig. 3b2 and d2). After incubation for 7 d, the distinctive prism-like structures strengthened and became more homogeneous on samples from the PASP-PEG-1.4 (Fig. 3a3) and PASP groups (Fig. 3c3). The prism-like structures on samples from the PASP-PEG-0.6 group remained unchanged at 7 d (Fig. 3b3) compared to that at 5 d (Fig. 3b1). However, the renewed layer was flatter and denser on samples from the DDW group at 7 d (Fig. 3d3). At high magnification, the surface morphology of the PASP-PEG-1.4 (Fig. 3a4) and PASP groups (Fig. 3c4) was quite different from that of the other two groups, with the wave-like microplates growing thicker and more compact as time increased. However, after incubation for 7 d, the minerals came out to deposit randomly on the PASP-PEG-0.6 (Fig. 3b4) and DDW group samples (Fig. 3d4), losing the microplate structures at 5 d (Fig. 3b2 and d2). These regenerated crystals were of much smaller size and nonuniform shape, which was even more obvious on samples from the DDW group. Meanwhile, the Ca/P ratios of the regenerated minerals measured by EDS at 7 d were 1.53, 1.26, 1.52 and 1.27 for the PASP-PEG-1.4, PASP-PEG-0.6, PASP and DDW groups, respectively (Fig. 3a4–d4). Moreover, the gel-like materials were observed as the yellow arrows indicated in Fig. 3a3–d3. The cross-sectional SEM images of remineralized enamel surfaces (Fig. 3a5−d5) showed that the newly regenerated layer of samples treated with PASP-PEG and PASP was tightly connected to its underlying natural enamel. The thickness of the regenerated layer in the PASP-PEG-1.4, PASP-PEG-0.6, PASP and DDW groups was 10.1 μm (Fig. 3a5), 7.0 μm (Fig. 3b5), 10.0 μm (Fig. 3c5), and 2.6 μm (Fig. 3d5), respectively.

**Fig. 4:**
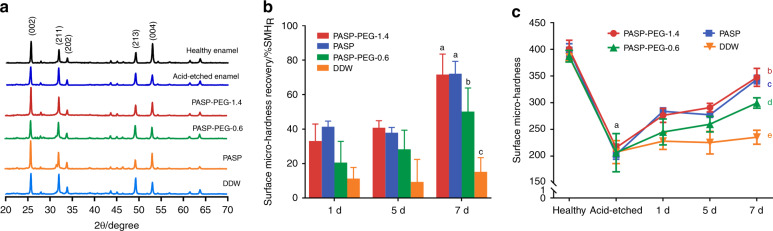
Crystal form and surface micro-hardness of regenerated minerals. **a** XRD spectrum of healthy tooth enamel, acid-etched enamel and tooth enamel treated with PASP-PEG-1.4, PASP, PASP-PEG-0.6 or DDW after incubation in 1.5× SBF for 7 d. **b** The surface micro-hardness of healthy enamel, acid-etched enamel, and acid-etched enamel treated with PASP-PEG-1.4, PASP, PASP-PEG-0.6 and DDW after immersion in 1.5 × SBF for 1, 5 and 7 d (mean ± SD, *n* = 5). **c** The surface micro-hardness recovery (%SMH_R_) of acid-etched enamel treated with PASP-PEG-1.4, PASP, PASP-PEG-0.6 and DDW after immersion in 1.5 × SBF for 1, 5 and 7 d (mean ± SD, *n* = 5). Dissimilar letters indicate significantly different values (*P* < 0.01)

The XRD patterns of regenerated crystals on enamel surfaces are shown in Fig. 4a. According to the standard card JCPDF #01-074-0565, the diffraction peaks appeared at 25.88, 31.77, 34.06, 49.49 and 53.22 degrees corresponding to the (002), (211), (202), (213) and (004) facets of the HA crystal, respectively. These peaks appeared on the healthy enamel as expected. After mineralization, all groups presented XRD patterns similar to that of healthy tooth enamel, indicating that the predominant component of the inorganic phase was HA.

The surface micro-hardness and surface micro-hardness recovery (%SMH_R_) results are shown in Fig. 4b, c (mean ± SD, *n* = 5). The hardness of healthy enamel and acid-etched enamel were almost the same among the four groups (Fig. 4b); 392.83 ± 12.07 and 207.97 ± 18.24, respectively. The surface micro-hardness of all groups increased with increasing time. After 7 d of immersion in 1.5 × SBF, the PASP-PEG-1.4 and PASP groups achieved the greatest increase in surface micro-hardness (Fig. 4b), raising the surface micro-hardness to 347.48 ± 15.03 and 343.39 ± 7.10, respectively, and the %SMH_R_ (Fig. 4c) was 71.64 ± 10.62 and 72.15 ± 6.36, respectively. The PASP-PEG-0.6 group produced an inferior effect compared with the above two groups, restoring the surface micro-hardness and %SMH_R_ to a moderate degree after 7 d of mineralization (surface micro-hardness: 299.46 ± 8.72, %SMH_R_: 50.18 ± 12.20). Nevertheless, the hardness of the DDW group increased slightly to 235.25 ± 11.79, and the %SMH_R_ value was only 15.17 ± 7.40 at 7 d.

### Water contact angle and anti-adhesion performance of PASP-PEG

Wettability was characterized according to the water contact angles by sessile drops of deionized water on the surfaces coated by different materials (Supplementary Fig. S3). HA slices coated with PASP-PEG exhibited high hydrophilicity, among which PASP-PEG-1.4-treated samples showed better hydrophilicity than PASP-PEG-0.6-treated samples, with contact angles of 14.3° and 20.0°, respectively. Samples from the PASP group exhibited a contact angle of 24.5°, rendering a higher surface hydrophilicity than that of samples from the DDW group, which exhibited a contact angle of 29.7°.

**Fig. 5:**
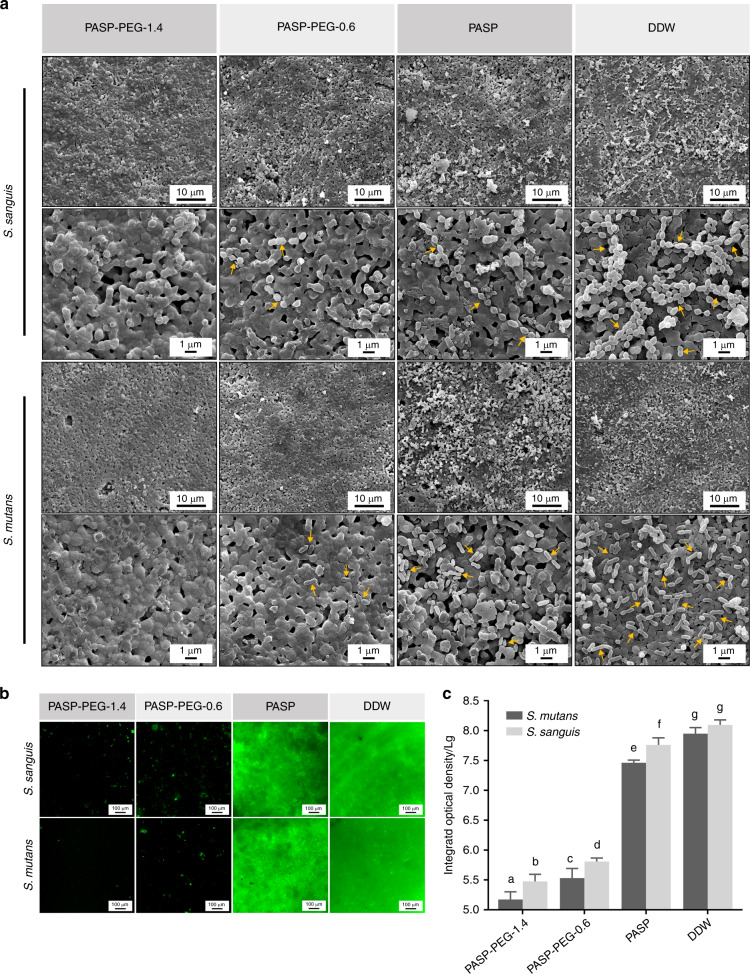
Anti-adhesion performance comparison among the HA slices coated with PASP-PEG-1.4, PASP-PEG-0.6, PASP and DDW. **a** SEM images of bacterial adhesion for 24 h. **b** Fluorescence microscope images of bacterial adhesion for 24 h. **c** Integrated optical density (IOD) values of fluorescence microscope images (mean ± SD, *n* = 5). Dissimilar letters indicate significantly different values (*P* < 0.01)

The effect of different coatings on the bacterial attachment was investigated by SEM (Fig. 5a). Bacteria could barely be seen on PASP-PEG-1.4-coated samples, and only several cells were scattered on PASP-PEG-0.6-coated samples. Bacteria forming long chains or evenly distributed on the surface could be observed on PASP-treated samples, rather than a large number of bacteria aggregating together or piling up on DDW-treated samples. The attachment of *Streptococcus sanguis* (*S. sanguis*) and *Streptococcus mutans* (*S. mutans*) was further tested using SYTO 9 staining after 24 h of incubation, and the results were in accordance with the SEM results. Almost no bacterial attachment was detected on the PASP-PEG treated groups, which was even more obvious on PASP-PEG-1.4 treated samples. In contrast, homogenous coverage of both *S. sanguis* and *S. mutans* was noticeable in DDW-treated samples (Fig. 5b). As shown in Fig. 5c, the integrated optical density (IOD) values (which grew exponentially and were log transformed) of PASP-PEG-coated samples were significantly lower than those of the other two groups (*P* < 0.01), and PASP-PEG-1.4-coated samples presented the lowest IOD values of both *S. sanguis* and *S. mutans* attachment (mean ± SD, *n* = 5). The bacterial attachment of PASP-coated samples was also less than that of DDW-coated samples, resulting in an appropriate decrease in IOD values (*P* < 0.01). In addition, more *S. sanguis* than *S. mutans* was adhered on the samples of each group. Significant differences were found in the PASP and PASP-PEG groups (*P* < 0.01).

## Discussion

The success to synthesize PASP-PEG was confirmed by ^1^H NMR and ATR-FTIR (Fig. 1a, b). The CCK-8 assay demonstrated the low cytotoxicity of PASP-PEG (Fig. 1c), which are appropriate for further biomedical applications.

Human teeth are surrounded by a moist and flowing liquid environment. To repair acid-etched enamel (with HA as the main component), the immobilization of inducers on the surface is highly necessary. To directly quantify the HA-binding capacity of PASP-PEG, we performed adsorption tests (Fig. 2). PASP-PEG adsorption on HA (Fig. 2a) has a very similar trend to other mineralization templates reported in previous studies.^22^ The adsorption amount increased rapidly at lower concentrations and then approached saturation as the concentration increased from 1.4 mg·mL^−1^ to 1.6 mg·mL^−^^1^. This could be explained by the earlier observations that the saturated absorption quantity depends on the concentrations of adsorbate solutions under the same experimental conditions.^23^ We then performed adsorption tests of PASP-PEG-1.4, PASP-PEG-0.6, PASP and PEG on acid-etched enamel samples (Fig. 2b). After being coated by different materials, the characteristic peaks of PASP-PEG, PASP and PEG were clearly detected, demonstrating the successful coating of materials on the enamel surface. Following deionized water washing, the characteristic peak disappeared in PEG-coated samples, indicating that very little PEG remained on the enamel surface. In contrast, the peaks of PASP-PEG and PASP on the tooth enamel samples were still strong after washing. This suggests that the adsorption capability of PASP-PEG and PASP on the acid-etched enamel surface is much higher than that of PEG, and the binding is strong enough to resist deionized water washing. These results are consistent with a previous study showing that PASP is capable of binding to HA^24^ and enamel surfaces.^25^ Its strong HA/tooth surface-binding ability is an important prerequisite to fulfill its remineralization function in the flowing liquid environment, which is probably attributed to the electrostatic interactions between the large number of negatively charged carboxylic groups of PASP-PEG and the positively charged calcium site (C site) on the HA surface.^24,25^

To better understand whether the concentration of PASP-PEG would affect remineralization, samples were also coated with PASP-PEG at a lower concentration (0.6 mg·mL^−1^). SEM images showed that samples coated by PASP-PEG-1.4 and PASP had distinctive prism-like structures (Fig. 3a1 and c1) and wave-like microplates highly interconnecting to form porous flower clusters after 5 d of mineralization (Fig. 3a2 and c2). After incubation for a longer time, the prism-like structures strengthened at 7 d (Fig. 3a3 and c3), and the microplates grew thicker and more compact (Fig. 3a4 and c4). Meanwhile, the Ca/P ratio of regenerated minerals at 7 d was 1.53 (Fig. 3a4) for the PASP-PEG-1.4 group, revealing that the regenerated crystal was similar to healthy human enamel (1.48)^26^ and HA (Ca/P 1.67).^27^ It has been confirmed experimentally that PASP has a similar role as mineralization proteins,^28–30^ which control the nucleation sites and conversion of amorphous calcium phosphate (ACP) into oriented apatite crystals.^28–31^ Carboxyl groups of mineralization proteins or organic molecules can interact with Ca ions to form a calcium complex.^32,33^ After acid etching, the formation of ACP offers more charged domains on the enamel surface.^34^ PASP with a high charge density interacts with ACP on the enamel surface and chelates the free calcium ions in the solution to form mineral-PASP complexes. These complexes can not only act as precursors of mineralization but also have a significant influence on mineral nucleation and orientation.^28,35,36^ In contrast, the samples from the DDW group demonstrated a relatively flat renewed layer at 5 d (Fig. 3d1 and d2), which became flatter (Fig. 3d3) at 7 d with the minerals depositing randomly on the surface (Fig. 3d4). In fact, the growth of new minerals in the DDW group at 5 d was mainly attributed to the charge adsorption effect between the free mineral ions and charged domains on the acid-etched enamel surface.^37^ As time increased, the remineralization got out of control since it lacked the regulation of PASP-PEG/PASP, resulting in the disordered aggregation and poor crystallinity of regenerated crystals, with the lowest enamel surface micro-hardness and %SMH_R_. Therefore, these findings demonstrated that PASP-PEG could act as a regulator with a similar function to acidic mineralization proteins. It is also notable that samples coated by PASP-PEG-0.6 showed prism-like structures (Fig. 3b1) and microplates (Fig. 3b2) at 5 d. Nevertheless, the minerals deposited randomly on the surface (Fig. 3b4), losing the microplate structure after incubation for a longer time. Previous studies have demonstrated that PASP could affect calcium phosphate mineralization in a concentration-dependent manner, including the kinetics of mineral formation and structure of the precursor phases, which then affect the structure and morphology of the final crystalline product.^38,39^ In addition, PASP-PEG-0.6 adsorbed onto the acid-etched surface provides fewer carboxyl groups than PASP-PEG-1.4, which means a weaker ability to form mineral-PASP complexes. The regulatory effect of PASP-PEG-0.6 decreased over time. As a result, PASP-PEG-0.6 had an inferior effect on the modulation of crystal growth compared to PASP-PEG-1.4. These findings also coincided with the cross-sectional SEM images. The thickness of the regenerated layer in the PASP-PEG-1.4, PASP-PEG-0.6, PASP and DDW groups was 10.1 μm (Fig. 3a5), 7.0 μm (Fig. 3b5), 10.0 μm (Fig. 3c5), and 2.6 μm (Fig. 3d5), respectively, demonstrating the excellent remineralization ability of PASP-PEG-1.4 during the whole process. In addition, gel-like materials were detected in the SEM image (Fig. 3a3–d3), which were confirmed to be ACP,^40^ as a consequence of the formation of amorphous gel-like material at supersaturations.^41^ ACP can be transformed into HA both in vitro and in vivo by prolonging the incubation time.^40^ In contrast to a previous study where the enamel hardness could be recovered to nearly the same as healthy enamel,^14,42^ the %SMH_R_ of the PASP-PEG-1.4 group was approximately 71.64, which is likely attributed to the presence of gel-like ACP in supersaturations.

The XRD patterns (Fig. 4a) showed that some characteristic peaks, such as (002) and (004), were weakened after acid etching. After incubation in 1.5× SBF for 7 d, the typical (002) and (004) peaks were clearly strengthened in the PASP-PEG-1.4 group and PASP group. The regenerated crystals of these two groups exhibited XRD patterns similar to those of healthy enamel. The sharp and intense (002) and (004) diffractions indicate that the newly formed crystals were oriented almost along the crystallographic c-axis.^43^ The ratio of the diffraction intensity of (002) to (211) was used to describe the orientation degree.^44^ From the XRD results, the ratios of the PASP-PEG-1.4 (1.66) and PASP groups (2.02) were obviously higher than those of the PASP-PEG-0.6 (1.15) and DDW groups (1.08), which demonstrated that the orientation degree of regenerated crystals was significantly improved in the presence of PASP-PEG/PASP. These results are consistent with previous work that, through an oriented attachment (OA) mechanism, PASP could induce the formation of ribbon-shaped apatite crystals oriented with their c-axis along the long axis of the fibril.^29^ Taken together, these findings support the hypothesis that PASP-PEG promotes the ordered precipitation of calcium phosphate, generating polycrystalline and oriented HA crystals. The concentration of PASP-PEG affected the adsorption efficiency and remineralization effect, and PASP-PEG-1.4 achieved a more satisfying effect than PASP-PEG-0.6.

*S. sanguis* is among the earliest colonizers of the human teeth and can be detected in high numbers in initial dental biofilms.^45^ *S. mutans*, widely recognized as a major cariogenic bacterium, can adhere to *S. sanguis* and cause dental caries, which may progress to pulpitis without proper treatments.^46^ Therefore, we investigated the anti-adhesive capacity of PASP-PEG against these streptococci. SYTO 9 can penetrate cells with intact and damaged membranes; thus, all bacteria that adhered to HA slices were labeled with green fluorescence. According to the SEM images (Fig. 5a), fluorescence microscopy images (Fig. 5b) and the IOD values (Fig. 5c) of the four groups, bacterial attachment of the PASP-PEG groups was significantly reduced compared to that of the PASP group and DDW group, demonstrating the excellent capacity of PASP-PEG to inhibit bacterial adhesion. A decrease in the contact angle values of the PASP-PEG groups indicated the higher hydrophilicity of the PASP-PEG group than that of the other two groups (Supplementary Fig. S3). Prior studies have demonstrated that hydrophobic interactions contribute to the initial bacterial adherence to solid surfaces.^47^ The bacterial resistance of PASP-PEG-modified surfaces is probably attributed to both hydration and steric hindrance effects.^18^ Each ethylene glycol unit in the PEG backbone can strongly bind to one water molecule and bridge the ether oxygen along the helical PEG chain,^48,49^ resulting in the formation of a highly hydrated layer that effectively reduces the hydrophobic interactions of bacteria with the HA surface. In addition, “steric repulsion” can be obtained from the long chain of PASP-PEG, which is an entropic effect concerning the change in free energy associated with confinement and the dehydration of soft polymer chains.^18^ Therefore, bacteria, whether live or dead, could be inhibited by attaching to the surfaces due to the low adherence of the PASP-PEG-coated HA slices. Several reports have shown that the percentage of dead cells reaches 70–80% in the inner layer at the initial stage of dental biofilm formation, which promotes the formation of plaque biofilm^50^ and may trigger inflammation/immune responses.^51^ Consequently, the anti-adhesive property of PASP-PEG is of great importance to resist subsequent bacterial aggregation and biofilm formation without triggering inflammation and immune responses, since the dead bacteria may still remain on the surface after being killed. Interestingly, the adhesion amount on DDW group samples was higher than that of PASP group samples (Fig. 5). According to Guan et al.^52^ the negatively charged PASP on the HA surface tends to repel negatively charged bacteria. It is also reasonable to postulate that the hydrophilicity of PASP, attributed to hydrophilic carboxy groups, is likely to contribute to this outcome. This is also confirmed by the previous water contact angle test that the contact angle value of the PASP group is smaller than that of the DDW group (Fig. S3). It is also worth noting that more *S. sanguis* than *S. mutans* adhered to the samples of each group, consistent with earlier observations that *S. sanguis* yielded stronger adhesion forces to the healthy or acid-etched enamel surfaces than *S. mutans*.^53^

Several protocols are recommended for the daily and clinical use of PASP-PEG. For daily oral healthcare, PASP-PEG could be provided through various home care products, such as toothpaste, mouthwashes, and varnish, during or immediately after oral hygiene procedures. Brushing teeth with toothpaste containing PASP-PEG helps effectively reduce bacterial adhesion afterwards. Rinsing the mouth or smearing teeth every day with mouthwashes or varnish containing PASP-PEG immediately after oral hygiene procedures could not only accelerate the remineralization of enamel but also reduce the follow-up bacterial adhesion during the whole day. For clinical treatment, a gel or varnish containing PASP-PEG with a relatively high concentration could be smeared onto the teeth to form a coating after the teeth are cleaned and dried with air flow. After that, mineralized solutions abundant in mineral ions can be applied to facilitate the repair of demineralized enamel and reduce dental biofilm accumulation. The above points will be considered in our future clinical trials. Therefore, PASP-PEG may be competitive for effective repair of demineralized enamel and may overcome the drawbacks of conventional clinical treatments.

There are still some limitations in our study. Future studies may consider remineralization and anti-adhesion conditions that more closely resemble the oral environment. For example, since the initial colonization of streptococci onto the tooth surface involves their attachment to the acquired pellicle, the potential of PASP-PEG to inhibit the adherence of bacteria to the acquired pellicle could be further evaluated.

**Fig. 6:**
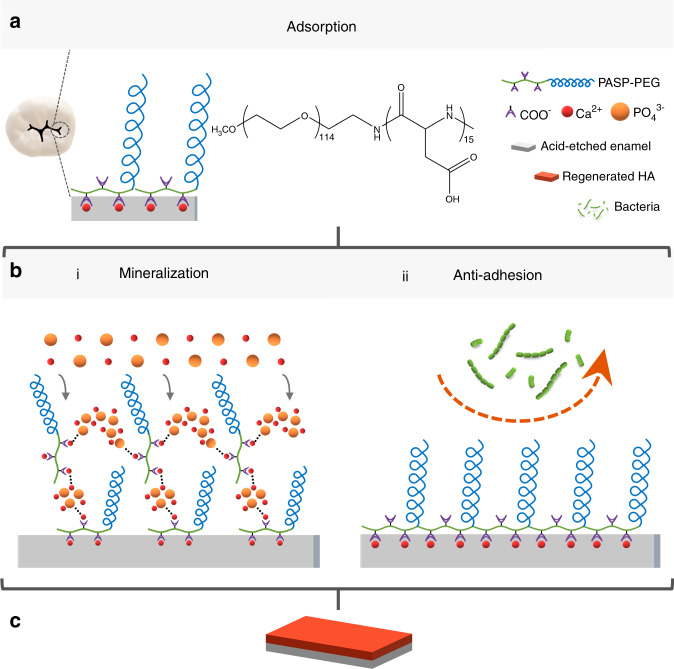
Schematic diagram of PASP-PEG in initial enamel caries treatment. **a** PASP-PEG interacts with calcium ions on the acid-etched enamel surface. **b** i PASP-PEG on the enamel surface chelates the free calcium ions and phosphate ions in the solution to induce in situ mineralization; ii PASP-PEG on the enamel surface forms a brush-like barrier to inhibit bacterial adhesion (*S. sanguis and S. mutans*). **c** Restoration of initial enamel caries is achieved via the remineralizing and anti-adhesive two-in-one strategy

Our research represents the first report about the application of synthetized PASP-PEG in demineralized enamel treatment. As summarized in Fig. 6, PASP-PEG possesses a high affinity for the acid-etched enamel surface, as the premise and foundation for the following two functions. (1) PASP-PEG could chelate the free mineral ions in the solution to realize in situ self-healing remineralization, effectively improving the mechanical properties of acid-etched enamel. (2) PASP-PEG could form a non-fouling coating that significantly reduces bacterial (*S. sanguis* and *S. mutans*) attachment.

## Materials and methods

### Synthesis of PASP-PEG

PASP-PEG was synthesized according to a modified method.^54^ b-Benzyl-l-aspartate NCA (BLA-NCA) was purchased from Shanghai Macklin Biochemical Co., Ltd. Other reagents and solvents were purchased from Tianjin Bodi Chemical Holding Company (analytical grade). N,N-Dimethylformamide (DMF) was dried and distilled before use, and other reagents were used as received. Poly(ethyleneglycol)-poly(b-benzyl-L-aspartate) (PEG-PBLA) was synthesized by ring-opening polymerization (ROP).^55^ First, BLA-NCA (2.88 g) was completely dissolved in DMF (12.90 mL). Afterwards, chloroform (114 mL) was added into the reaction flask. Then, CH_3_-PEG-NH_2_ (1.20 g) in chloroform (15 mL) was added into the solution. The reaction mixture was stirred for 72 h at 35 °C under a mild flow of nitrogen. The resulting solution was precipitated into cold diethyl ether and then centrifuged (5 000 r·min^−1^, 5 min) to collect the precipitate. Finally, the precipitate was dried under vacuum.

To obtain PASP-PEG, PEG-PBLA (3.90 g) was first dispersed in chloroform (90 mL). Then, NaOH (0.43 mol·L^−1^, 115 mL) was added to the solution, which was prepared with a mixed solvent of water, methanol and isopropanol in a volume ratio of 1:2:2. After stirring for 10 min at 0 °C, the mixed solution was neutralized with acetic acid. The mixed solution was then concentrated by rotary evaporation and dialyzed against water for 24 h. Finally, PASP-PEG was obtained after lyophilization.

### Characterization of PASP-PEG

^1^H NMR spectra of PASP-PEG were recorded on a Bruker 400 MHz spectrometer (AV III HD 400 MHz, Germany) at room temperature with tetramethylsilane (TMS) as an internal standard, using D_2_O as the solvent. ATR-FTIR spectra were recorded using a spectrometer (Nicolet iS10, Thermo Scientific, USA).

### Cytotoxicity assay

A CCK-8 assay was performed on human oral keratinocytes (HOKs) to evaluate the cytotoxicity of PASP-PEG. HOKs were cultured in Minimum Essential Media α (α-MEM) (Gibco, Thermo Scientific, USA) supplemented with 10% fatal bovine serum (FBS) (Gibco, Thermo Scientific, USA) and 1% penicillin/streptomycin at 37 °C in 5% CO_2_ with 95% relative humidity. The HOKs were seeded in a 96-well microtiter plate at a density of 2 × 10^4^ cells per well and incubated for 24 h. The culture media was replaced with 100 µL fresh culture media containing different concentrations of PASP-PEG ranging from 0.6 mg·mL^−1^ to 1.6 mg·mL^−1^. After incubation for another 24 h, the culture media was replaced with 100 µL α-MEM containing 10% CCK-8 (Dojindo, Japan) per well, and the plates were incubated at 37 °C for 3 h. The absorbance of each well was measured using a microplate reader (Spectra Plus, Tecan, Zurich, Switzerland) at a wavelength of 450 nm. The cell viability (%) was calculated using the following equation: 100 × ([A]test – [A]blank)/([A]control – [A]blank), where [A]test, [A]control, and [A]blank are the absorbance values of the wells with PASP-PEG, without PASP-PEG, and without PASP-PEG and cells, respectively. For each sample, the absorbance was the average value measured from five wells in parallel.

### Preparation of tooth enamel samples

Bovine incisors free of lesions and cracks were collected. The periodontal membrane and dental calculus were removed. The incisors were cut with a diamond saw (Minitom, Struers, Copenhagen, Denmark) longitudinally and embedded in acrylic resin to obtain enamel plates. The surfaces of the samples were polished with 800-, 1 500-, 2 000-, and 2 400-grit carbide-polishing papers under running water and were partly painted with acid-resistant nail varnish, leaving an exposed 5 mm × 5 mm window. The polished specimens were ultrasonicated (FS20, Fisher Scientific, Pittsburgh, PA, USA) for 10 min to remove the smear layer. The original surface micro-hardness (Vickers hardness, HV) was measured using a Vickers hardness tester (MMT-X7A, Matsuzawa, Japan) with a diamond indenter under a 50 gf load for 10 s.^56^ Each sample was given five indentations, and the average value was calculated. Enamel samples with baseline surface micro-hardness of more than 300 were included in the following steps. Demineralized models were prepared based on published procedures.^57^ In brief, each sample was immersed in phosphoric acid (37%, 10 mL) for 45 s, washed with phosphate-buffered saline (PBS, pH 7.4) four times and sonicated for 5 min. The samples were stored at 4 °C in 0.05% thymol solution before use.

### Adsorption of PASP-PEG on HA powder and tooth enamel

The optimum absorption wavelength was obtained by full wavelength scanning of standard samples through UV-Vis (UH5300, Hitachi, Japan). A standard curve was obtained by scanning PASP-PEG with a series of concentrations at the optimum absorption wavelength. HA powder (medical grade, spherical HA powder, 10 μm in diameter) was purchased from the National Engineering Research Center for Biomaterials, Sichuan University. HA powder (25 mg) and PASP-PEG solutions (1 mL) with concentrations ranging from 0.2 to 1.6 mg·mL^−1^ were added to 2 mL polyethylene tubes and gently shaken at 37 °C for 24 h. The tubes were then centrifuged (12 000 *g*, 3 min) to collect the supernatants, and the concentrations were determined by UV absorbance analysis (UH5300, HITACHI, Japan) at a wavelength of 288 nm. The total mass of PASP-PEG adsorbed on the HA powder was calculated by the decrease in HA in the solution, according to the following equation.^42^

$$A = x/m = v(c_0 - c)/m$$

*A* represents the amount of PASP-PEG adsorbed per mass unit of HA. *x* represents the total mass of PASP-PEG adsorbed on the HA powder, and *m* is the mass of the HA powder. *c*_0_ and *c* represent the concentration of PASP-PEG before and after adsorption, respectively. *v* is the volume of PASP-PEG solution. The mean values of three measurements were recorded for each concentration.

Enamel samples were randomly divided into 4 groups, PASP-PEG-1.4, PASP-PEG-0.6, PASP and PEG (100 µL) were evenly pipetted onto the enamel window area. After drying naturally at room temperature, each sample was rinsed with deionized water three times gently and dried again. ATR-FTIR (Nicolet iS10, Thermo Scientific, USA) characterization was performed on the window area of each sample before and after coating, as well as after washing with deionized water.

### Remineralization of acid-etched tooth enamel in vitro

The acid-etched enamel samples were randomly divided into four groups coated with PASP-PEG-1.4, PASP-PEG-0.6, PASP and DDW, respectively.^57^ In brief, different solutions of 100 µL were evenly pipetted onto the window area. After drying naturally at room temperature, each sample was rinsed with deionized water three times gently and dried again. Then, 1.5× SBF was prepared according to a previously reported method.^58^ Each coated sample was vertically placed in a 15 mL polyethene tube and immersed in 10 mL 1.5× SBF. All tubes were stored in an incubator at 37 °C. The 1.5× SBF was replaced with fresh solution every day. After 1, 5 and 7 d, the samples were collected and rinsed with deionized water three times before being dried for further characterization.

### Analysis of the in situ regenerated crystal on tooth enamel

The morphology of regenerated crystals was analyzed using SEM (Inspect F50, FEI, USA). The surfaces were sputter-coated with Au before observation. EDS (INCA350, Oxford, UK) was applied to measure the Ca/P ratio of the regenerated minerals. XRD (Ultima IV, Rigaku, Japan) with Cu−Ka radiation (40 kV, 110 mA) was carried out to examine the crystal form of the regenerated minerals. The surface micro-hardness was measured using a Vickers hardness tester (MMT-X7A, Matsuzawa, Japan) under a 50 gf load for 10 s. Each sample was analyzed five times. The surface micro-hardness of healthy enamel and demineralized enamel without any treatment was also measured. The SMH (original surface micro-hardness), SMH_1_ (after acid erosion), and SMH_2_ (after mineralization) were recorded after 1, 5 and 7 d of remineralization. The degree of surface micro-hardness recovery (%SMH_R_) was calculated as %SMH_R_ = 100 × (SMH_2_ – SMH_1_)/(SMH – SMH_1_).^59^

### Water contact angle measurement

The HA slices (ф8 × 2 mm, National Engineering Research Center for Biomaterials, Sichuan University) were divided into four groups and coated with PASP-PEG-1.4, PASP-PEG-0.6, PASP and DDW as previously described. The contact angles of the coating samples were recorded using the sessile drop method (Drop Shape Analyser-DSA25, Kruss, Germany).^60^ Ultrapure distilled water (1 μL) was used as the working fluid.

### Bacterial adhesion

*S. sanguis* and *S. mutans* were used to evaluate the anti-adhesive performance of different coatings. The HA slices were placed into 24-well cell culture plates after being sterilized and then immersed in sterile PASP-PEG-1.4, PASP-PEG-0.6, PASP or DDW (1 mL) for 2 h at 37 °C. Then, coated samples were transferred to new 24-well cell culture plates with freshly prepared bacterial suspensions, of which the concentrations were spectrophotometrically adjusted to the same (OD_600_ = 0.5). After incubating at 37 °C for 24 h, the samples were rinsed with deionized water and stained with SYTO 9 fluorescent staining agent (Molecular Probes, Eugene, OR, USA) at room temperature for 15 min. All the stained surfaces were observed using a fluorescence microscope (Ti-U, Nikon) with a magnification of 40×. The image collection gates were set at 500–540 nm for SYTO 9. Five samples for each group were observed. The fluorescence images were analyzed quantitatively using threshold analysis of ImageJ software (ImageJ 1.52, Rawak Software Inc., Stuttgart, Germany). The integrated optical density (IOD) of each fluorescence image was calculated.

Bacterial attachment on HA surfaces was further analyzed by SEM. Bacteria were fixed with 2.5% (v/v) glutaraldehyde and dehydrated in graded alcohol (ranging from 30 to 100% (v/v)). The surfaces were sputter-coated with Au before observation.^61^

### Statistical analysis

All experimental data were checked for normal distribution and quality of variance with the Kolmogorov–Smirnov test and modified Levene test, respectively. One-way and two-way analyses of variance (ANOVA) were performed to detect the significant effects of the variables. Tukey’s multiple comparison tests were used for post hoc comparisons. Statistical significance for all tests was set at *α* = 0.05.

## Supplementary information

Supplementary information
