# Supplementary material for: Two-in-one strategy: a remineralizing and anti-adhesive coating against demineralized enamel
Source: Int J Oral Sci. 2020 Sep 29;12:27. doi: 10.1038/s41368-020-00097-y (PMC7524769; doi:10.1038/s41368-020-00097-y)

**Fig. S1 a** The absorbing curve of PASP-PEG. **b** The standard curve of PASP-PEG (R^2^=0.9985).


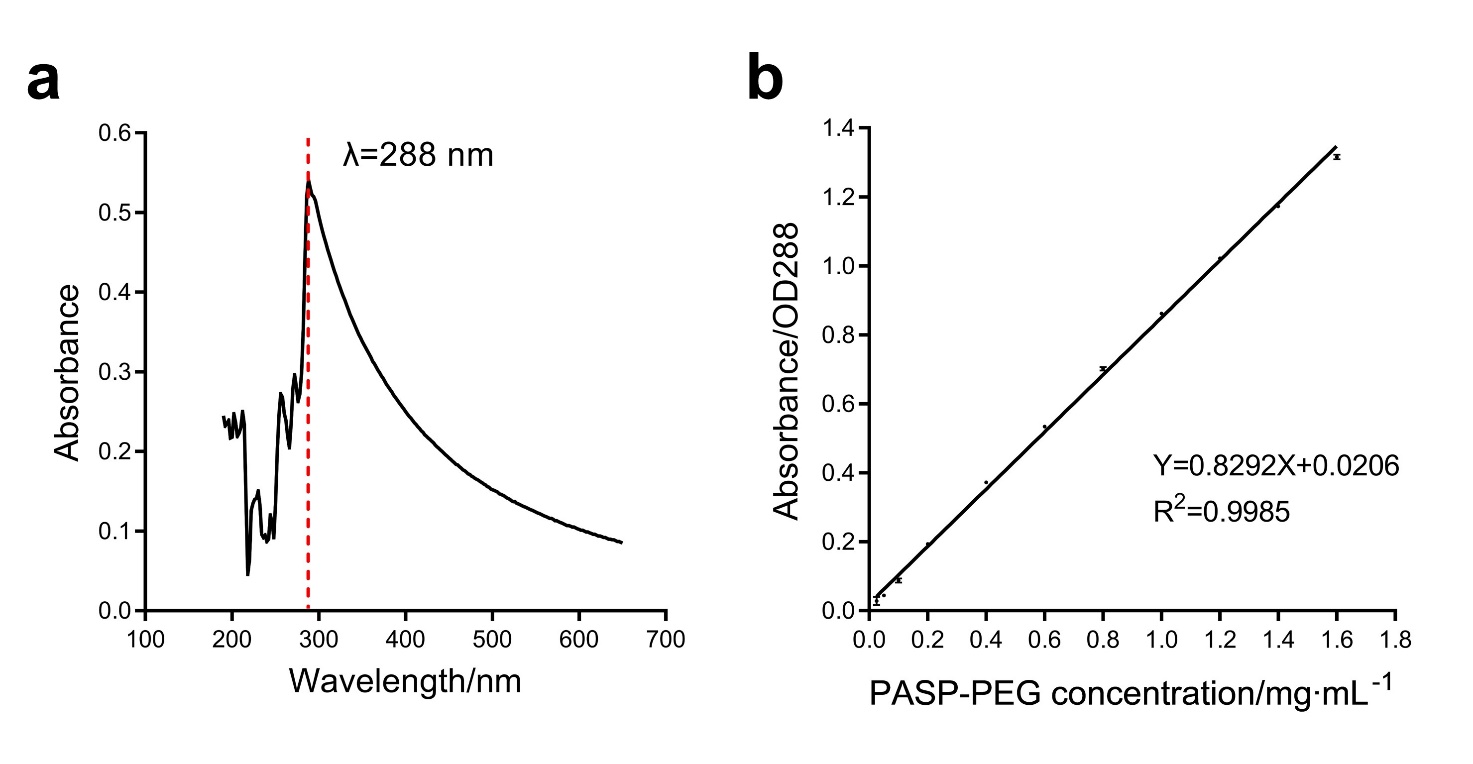
**Fig. S2** SEM images of acid-etched enamel surface. The inset is an enlargement showing greater detail.

**
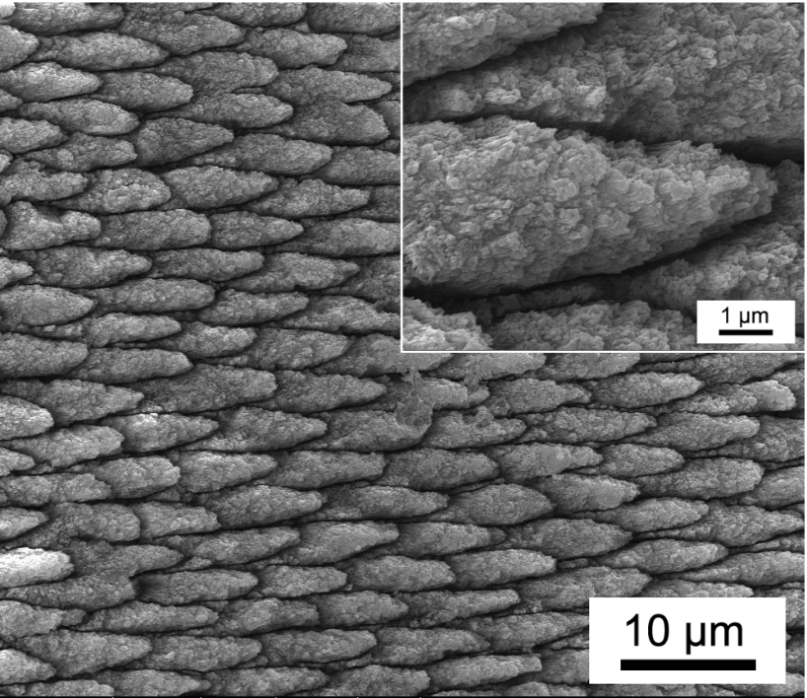
**

**Fig. S3** Water contact angle images of HA slices coated with PASP-PEG-1.4, PASP-PEG-0.6, PASP and DDW.


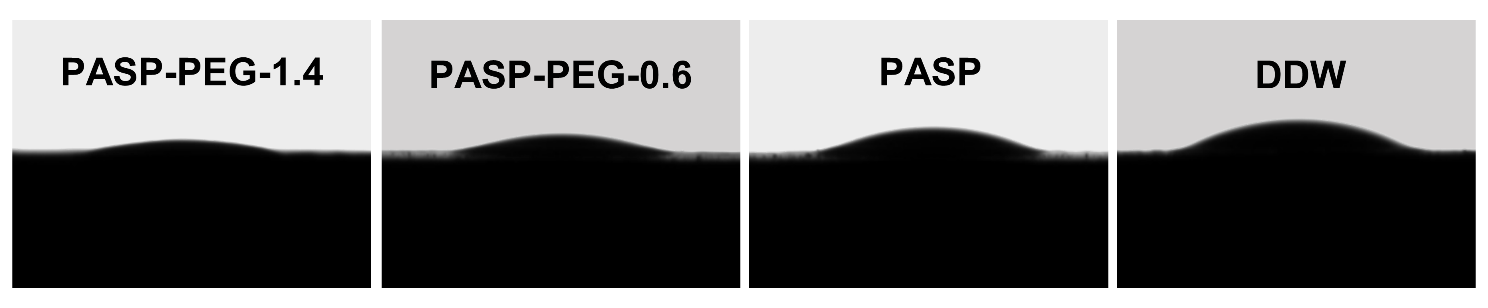

Supplement: Supplementary file 1 — Supplementary information [file 41368_2020_97_MOESM1_ESM.docx]
